# Effect of causative genetic variants on atherosclerotic cardiovascular disease in heterozygous familial hypercholesterolemia patients

**DOI:** 10.3389/fcvm.2023.1182554

**Published:** 2023-07-19

**Authors:** Anthony Matta, Jean Pierre Rabès, Dorota Taraszkiewicz, Didier Carrié, Jérôme Roncalli, Jean Ferrières

**Affiliations:** ^1^ Department of Cardiology, Civilians Hospital of Colmar, Colmar, France; ^2^ Department of Cardiology, Notre Dame des Secours University Hospital Center, Byblos, Lebanon; ^3^ School of Medicine and Medical Sciences, Holy Spirit University of Kaslik, Jounieh, Lebanon; ^4^ Department of Biochemistry and Molecular Genetics, Ambroise Paré University Hospital (APHP), Université Paris-Saclay, Paris, France; ^5^ UFR (Unité de Formation et de Recherche) Simone Veil-Santé, Versailles-Saint-Quentin-en-Yvelines University, Paris, France; ^6^ Department of Cardiology, Toulouse University Hospital, Rangueil, France; ^7^ Department of Epidemiology, Health Economics and Public Health, UMR INSERM 1295, Toulouse-Rangueil University Hospital, Toulouse University School of Medicine, Toulouse, France

**Keywords:** heterozygous familial hypercholesterolemia, genetic variant, cardiovascular disease, LDL-c, atherosclerosis

## Abstract

**Background:**

Heterozygous familial hypercholesterolemia (HFH) is an autosomal dominant genetic disorder leading to a lifetime exposure to high low-density lipoprotein cholesterol (LDL-c) level and an increased risk of premature atherosclerotic cardiovascular disease (ASCVD). We evaluate the effect of a causative genetic variant to predict ASCVD in HFH patients undergoing treatment.

**Materials and methods:**

A retrospective cohort was conducted on 289 patients with possible, probable, and definite diagnosis of HFH according to Dutch Lipid Clinic Network Score and in whom DNA analyses were performed and mean LDL-c level was above 155 mg/dl. The study population was divided into groups based on the presence or not of a causative variant (pathogenic or likely pathogenic). We observed each of the study’s participants for the occurrence of ASCVD.

**Results:**

A causative variant was detected in 42.2% of study participants, and ASCVD has occurred in 21.5% of HFH patients. The incidence of ASCVD (27% vs. 17.4%, *p* = 0.048) and the mean of LDL-c under an optimal medical treatment (226 ± 59 mg/dl vs. 203 ± 37 mg/dl, *p* = 0.001) were higher in HFH-causative variant carriers than others. After adjusting on confounders, ASCVD was positively associated with LDL-c level [OR = 2.347; 95% (1.305–4.221), *p* = 0.004] and tends toward a negative association with HDL-c level [OR = 0.140; 95% (0.017–1.166), *p* = 0.059]. There is no more association between the detection of a causative variant and the occurrence of ASCVD [OR = 1.708; 95% (0.899–3.242), *p* = 0.102]. Kaplan Meier and log rank test showed no significant differences in event-free survival analysis between study groups (*p* = 0.523).

**Conclusion:**

In this study population under medical care, it seems that the presence of a causative variant did not represent an independent predictor of adverse cardiovascular outcomes in HFH patients, and LDL-c level played an undisputable causal role.

## Introduction

Heterozygous familial hypercholesterolemia (HFH) is an autosomal dominant genetic disorder leading to a lifetime exposure to high low-density lipoprotein cholesterol (LDL-c) level and an increased risk of premature atherosclerotic cardiovascular disease (ASCVD). In most countries, the heterozygous form of familial hypercholesterolemia (FH) usually affects 1 in 313 to 120 individuals (1–3). The Dutch Lipid Clinic Network Score (DLCNS) is a valid diagnostic score for FH. It includes a set of criteria: patient’s family history of early-onset cardiovascular disease in his first-degree relatives, personal history of cardiovascular disease, physical signs of hypercholesterolemia (tendinous xanthoma and/or arcus cornealis prior to age 45 years), circulating level of LDL-c, and positive DNA analysis for a genetic variant in *LDLR* (low-density lipoprotein cholesterol receptor), *APOB* (apolipoprotein B), or *PCSK9* (proprotein convertase subtilisin/kexin type-9) gene (4). DLCNS stratifies the diagnosis of FH into four categories: unlikely (<3 points), possible (3–5 points), probable (6–8 points), and definite (>8 points). Thus, DNA testing is recommended in FH patients by several international and scientific societies. It ensures a precise molecular diagnosis, a screening cascade identifying unknown and asymptomatic FH patients among closed family members, an early initiation of optimal medical therapy, and a prognostic stratification (5, 6). Apart from the traditional HFH-causing variants in *LDLR*, *APOB*, and *PCSK9* genes, the detection of mutant *APOE* gene in HFH patients is recently considered as a cause or an exacerbating factor of HFH phenotype (6–8). It seems that patients with digenic causality, combined *LDLR* and *PCSK9* gene variants, experienced poor cardiovascular outcomes marked by a high frequency of non-fatal myocardial infarction (9). It is noteworthy that the risk of obstructive coronary artery disease in HFH patients with pathogenic variant and LDL-c level of ≥190 mg/dl was 22 times higher than that of general population with LDL-c level of ≤ 130 mg/dl (10). It was also six times higher in HFH patients without pathogenic variant compared with the reference group (10). In the setting of HFH, clinical trials evaluating the risk of atherosclerosis depending on DNA analysis are scarce in literature. Most published ones assess the difference in risk between FH patients and the general population. The present study compares cardiovascular outcomes in HFH population under medical care with versus without a causative variant and evaluates the association between different gene variants and ASCVD.

## Materials and methods

### Study design and population

A retrospective cohort was conducted on 854 patients who were referred to the Department of Preventive Cardiology at Toulouse University Hospital, Rangueil, France, and for whom the results of DNA analysis test are available. We collected the available controls of lipid panel during the follow-up period while receiving the maximum tolerated medical therapy. The follow-up period extended from the date of the first lipid panel till the occurrence of ASCVD or the last available follow-up. The DLCNS and means of collected LDL-c levels in the course of time were calculated for each of the study’s participants. Patients aged above 18 years old and who fulfilled the diagnostic criteria of possible, probable, and definite HFH according to DLCNS were included in this study (289 patients). Patients with incomplete data (lack of follow-up information), younger than 18 years old, with mean LDL-c of <155 mg/dl, and unlikely for HFH diagnosis (DLCNS of <3) were excluded from this study (558 patients). One patient with homozygous FH was also excluded. Six patients with genetic variant of unknown significance were excluded. Then, we observed study participants till the occurrence of a significant atherosclerotic cardiovascular event or the last available follow-up. The study population was divided into two groups: first, according to the development or not of ASCVD and, second, according to the detection or not of a causative variant. We evaluate the differences in the incidence of ASCVD and means of total cholesterol, LDL-c, HDL-c, triglycerides, lipoprotein(a) [Lp(a)], apolipoprotein A1 (Apo A1), and apolipoprotein B (Apo B) levels among the study groups.

### Data collection and end point

Baseline characteristics of study population, results of DNA analysis, and full lipid panel tests [total cholesterol, LDL-c, HDL-c, triglycerides, Lp(a), Apo A1, and Apo B] under an optimal tolerated lipid-lowering therapy were collected throughout the follow-up period. The DLCNS and means of cholesterol, LDL-c, HDL-c, triglycerides, Lp(a), Apo A1, and Apo B levels were calculated for each of study’s participants, respectively. ASCVD was defined by a more than 50% reduction in the diameter of peripheral arteries or carotids on Doppler ultrasound, an ischemic stroke was revealed on cerebral imaging, and more than 50% reduction in the coronary artery lumen was detected on a coro scanner or coronary angiography. DNA sequencing of the *LDLR*, *PCSK9*, *APOB*, and *APOE* genes were performed. All genetic variants and their causal effects were verified by “RJP” and subsequently classified as pathogenic, likely pathogenic, variant of unknown significance, likely benign, or benign. Considering their consequences, pathogenic and likely pathogenic causative genetic variants were segregated into two subtypes: moderate or severe. Severe variants encompass large rearrangements and point mutations accounting for non-sense, frameshifts, splicing, and initiation codon loss mutations. Moderate variants include missense, in-frame deletion, or duplication and 5′ regulatory mutations. The HFH-causative variant carriers group includes pathogenic and likely pathogenic variants, whereas HFH-no causative variant group includes variants of unknown significance, benign and likely benign variants, and patients with undetected genetic variant. We aim to evaluate if HFH with a causative variant patient undergoing medical care was more associated with ASCVD compared with HFH-no causative variant carrier. Patients were informed at hospital admissions that their clinical data could be used for research purposes in anonymous form, and non-opposition consent forms were obtained. The cohort was registered by the Ministry of Research and the Regional Health Agency Occitanie (no. DC-2017-298).

### Statistical analysis

Statistical analyses were performed using SPSS version 20.0. Qualitative variables were expressed by frequency and percentages, while quantitative variables were summarized as means and standard deviations. Categorical variables were compared with the use of *χ*^2^ test or Fisher’s exact test as appropriate, while continuous variables were studied with the use of *t*-test. Normality and variance homogeneity for continuous variables were checked. Kaplan–Meier curve and log rank test were used for survival analysis. Multivariable logistic regression analysis was used to test the association of ASCVD with HFH-causative variants. A two-sided *p*-value of ≤0.05 was considered to be of statistical significance.

## Results

**Figure 1:**
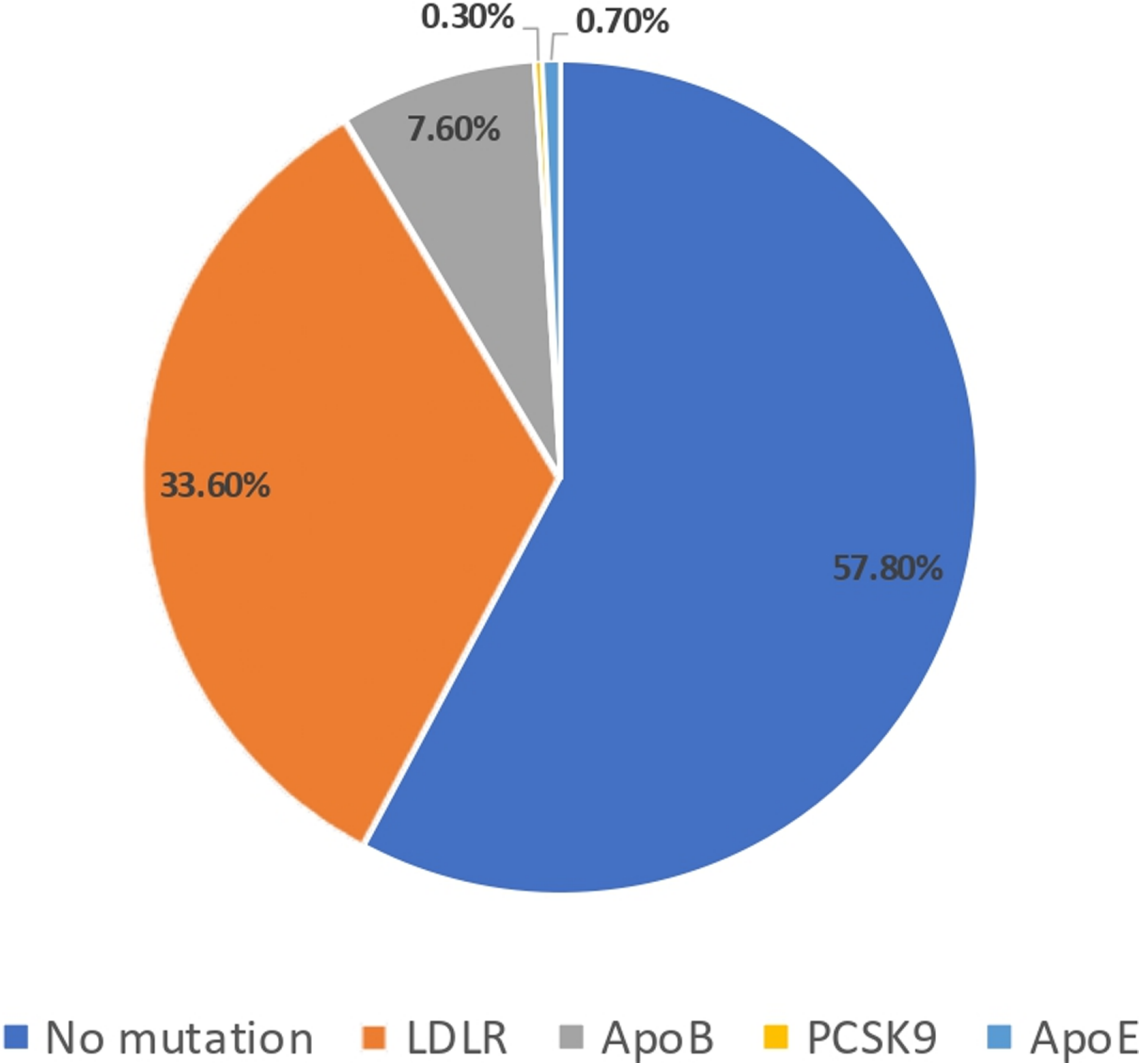
Pie chart representing the prevalence of no mutation, *LDLR*, Apo B, PCSK9, and Apo E mutations in the study population.

**Figure 2:**
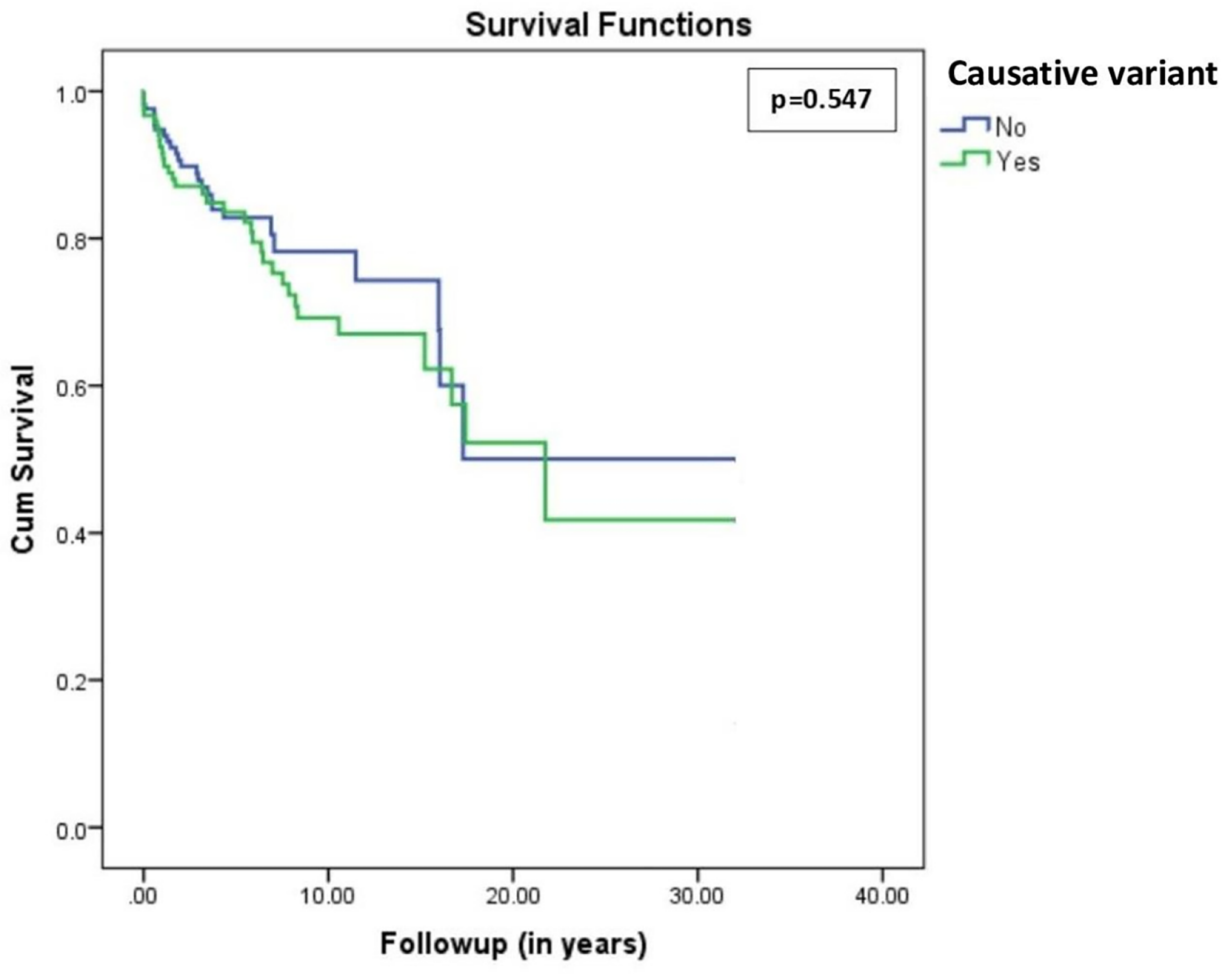
Kaplan–Meier survival analysis for freedom of atherosclerotic cardiovascular disease in FH patients with versus without causative mutation (*p* = 0.547).

Out of 854 screened patients, a total of 289 patients were included in this study. The mean age of study population was 49 ± 13 years old, and 37% of study participants were males. Based on DLCNS, the diagnosis of HFH was definite (>8 points) in 48.8%, probable (6–8) in 4.2%, and possible (3–5) in 47.1%. The DNA analysis detected a genetic causative variant in 42.2% of study participants. The causative variants were found on *LDLR* gene in 33.6%, *APOB* gene in 7.6%, *PCSK9* in 0.3%, and *APOE* in 0.7% (Figure 1). Over a mean follow-up period of 5.97 ± 5.97 years, ASCVD has occurred in 21.5% of study participants. The observed cardiovascular events were coronary artery disease (18.2%), ischemic stroke (1.4%), and peripheral artery disease (1.4%). Compared with no ASCVD group, HFH patients who developed ASCVD were commonly males (48.4% vs. 33.9%, *p* = 0.037), were older (52 ± 12 vs. 48 ± 13, *p* = 0.027), and had higher means of total cholesterol (310 ± 74 vs. 288 ± 46, *p* = 0.029) and LDL-c (233 ± 66 vs. 207 ± 41, *p* = 0.004) levels. In addition, causative variants were significantly more expressed in study participants with versus without ASCVD (53.2% vs. 39.2%, *p* = 0.048) (Table 1). On the other hand, HFH-causative variant carriers were younger (45 ± 14 vs. 52 ± 11, *p* = 0.001) with higher mean LDL-c level (226 ± 59 vs. 203 ± 37, *p* = 0.001). Also, HFH patients with pathogenic and likely pathogenic variant were at greater risk of ASCVD (27% vs. 17.4%, *p* = 0.048) (Table 2). After adjusting on confounders (age, sex, HDL-c and LDL-c levels), the multivariable logistic regression showed a positive association of LDL-c level [OR = 2.658; 95% (1.495–4.729), *p* = 0.001] and age [OR = 1.034; 95% (1.009–1.060), *p* = 0.008] with ASCVD, respectively. The HDL-c level tends toward a negative association with ASCVD [OR = 0.132; 95% (0.016–1.073), *p* = 0.058], whereas male sex tends toward a positive association [OR = 1.823; 95% (1.974–3.413), *p* = 0.061]] (Table 3—model 1). Unlike the results of bivariate analyses, the detection of a causative variant becomes no more significantly associated with ASCVD [OR = 1.713; 95% (0.902–3.256), *p* = 0.100] (Table 3—model 2). Only after excluding LDL-c from the statistical model, the association between the presence of causative genetic variant and ASCVD was statistically significant [OR = 2.149; 95% (1.170–3.948), *p* = 0.014] (Table 3—model 3). Similar results were found after a second revision of genetic analyses stratifying study participants into three categories: severe, moderate, and no causative genetic variant (Supplementary Tables S1 and S2). Note that the means of LDL-c differed significantly among study sub-groups (Table 4) and this difference was mainly observed between *LDLR* variant carriers and those with no causative variant (*p* = 0.003) (Table 5). Lastly, the Kaplan–Meier curve and log rank test failed to detect a significant difference in survival analysis for freedom of ASCVD between study groups (no causative variant vs. causative variant carriers, *p* = 0.547) (Figure 2).

**Table 1:** Characteristics of study population with versus without atherosclerotic cardiovascular disease (ASCVD).

	Study population (*N* = 289)	ASCVD group (*N* = 62)	No ASCVD group (*N* = 227)	*p*-Value
Age (year)	49 ± 13	52 ± 12	48 ± 13	0.027
Males (*N*, %)	107 (37%)	30 (48.4%)	77 (33.9%)	0.037
BMI (kg/m^2^)	24.3 ± 4.5	24.6 ± 3.9	24.2 ± 4.6	0.554
Smoker (*N*, %)	53 (18.3%)	11 (17.7%)	42 (18.5%)	0.891
Systemic hypertension (*N*, %)	44 (15.2%)	11 (17.7%)	33 (14.5%)	0.534
Diabetes mellitus (*N*, %)	8 (2.8%)	4 (6.5%)	4 (1.8%)	0.068
Causative mutation (*N*, %)	122 (42.2%)	33 (53.2%)	89 (39.2%)	0.048
Mutated gene (*N*, %)				0.163
*LDLR*	97 (33.6%)	25 (40.3%)	72 (31.7%)	
Apo B	22 (7.6%)	7 (11.3%)	15 (6.6%)	
PCSK9	1 (0.3%)	0	1 (0.4%)	
Apo E	2 (0.7%)	1 (1.6%)	1 (0.4%)	
DLNCS (*N*, %)				0.001
3–5 points (possible)	136 (47.1%)	13 (21.0%)	123 (54.2%)	
6–8 points (probable)	12 (4.2%)	11 (17.7%)	1 (0.4%)	
>8 points (definite)	141 (48.8%)	38 (61.3%)	103 (45.4%)	
Mean total cholesterol (mg/dl)	292 ± 54	310 ± 74	288 ± 46	0.029
Mean LDL-c (mg/dl)	213 ± 49	233 ± 66	207 ± 41	0.004
Mean HDL-c (mg/dl)	60 ± 25	56 ± 16	62 ± 27	0.162
Mean triglyceride (mg/dl)	125 ± 55	133 ± 58	123 ± 54	0.146
Mean Lp(a) (mg/dl)	44 ± 49	49 ± 43	43 ± 48	0.446
<10 mg/dl (%)	33%	26.3%	34.4%	0.589
10–50 mg/dl (%)	33%	34.2%	32.8%	
>50 mg/dl (%)	34%	39.5%	32.8%	
Mean Apo A1 (mg/dl)	156 ± 25	151 ± 22	157 ± 25	0.168
Mean Apo B (mg/dl)	150 ± 33	152 ± 39	149 ± 34	0.667
Follow-up (year)	5.97 ± 5.97	5.10 ± 6.36	6.20 ± 5.85	0.197

BMI, body mass index; *LDLR*, low-density lipoprotein cholesterol receptor; PCSK9, proprotein convertase subtilisin/kexin type-9; Apo, apolipoprotein; DLNCS, Dutch Lipid Network Clinic Score; LDL-c, low-density lipoprotein cholesterol; HDL-c, high-density lipoprotein cholesterol; Lp(a), lipoprotein (a).

**Table 2:** Characteristics of study population with versus without causative mutation.

	Study population (*N* = 289)	Causative mutation group (*N* = 122)	No causative mutation group (*N* = 167)	*p*-Value
Age (year)	49 ± 13	45 ± 14	52 ± 11	0.001
Males (*N*, %)	107 (37%)	46 (37.7%)	61 (36.5%)	0.838
BMI (kg/m^2^)	24.32 ± 4.46	24.09 ± 5.09	24.48 ± 3.97	0.470
Smoker (*N*, %)	53 (18.3%)	24 (19.7%)	29 (17.4%)	0.617
Systemic hypertension (*N*, %)	44 (15.2%)	14 (11.5%)	30 (18%)	0.129
Diabetes mellitus (*N*, %)	8 (2.8%)	4 (3.3%)	4 (2.4%)	0.725
Mean total cholesterol (mg/dl)	292 ± 54	299 ± 66	288 ± 42	0.087
Mean LDL-c (mg/dl)	213 ± 49	226 ± 59	203 ± 37	0.001
Mean HDL-c (mg/dl)	60 ± 25	57 ± 15	63 ± 30	0.032
Mean triglyceride (mg/dl)	125 ± 55	110 ± 41	137 ± 61	0.001
Mean Lpa (mg/dl)	44 ± 49	41 ± 44	46 ± 52	0.405
<10 mg/dl (%)	33%	32.6%	33.3%	0.532
10–50 mg/dl (%)	33%	36.8%	30.4%	
>50 mg/dl (%)	34%	30.5%	36.3%	
Mean Apo A1 (mg/dl)	156 ± 25	152 ± 27	159 ± 23	0.033
Mean Apo B (mg/dl)	150 ± 35	156 ± 41	145 ± 29	0.030
ASCVD (*N*, %)	62 (21.5%)	33 (27%)	29 (17.4%)	0.048
Follow-up (year)	5.97 ± 5.97	7.31 ± 6.54	4.98 ± 5.32	0.001

BMI, body mass index; LDL-c, low-density lipoprotein cholesterol; HDL-c, high-density lipoprotein cholesterol; Lp(a), lipoprotein (a); Apo, apolipoprotein; ASCVD, atherosclerotic cardiovascular disease.

**Table 3:** Statistical models of multivariable logistic regression investigating the association between the presence of causative mutation and development of atherosclerotic cardiovascular disease adjusted on confounders.

Model 1			
	OR	95% CI	*p*-Value
Sex	1.823	(1.974–3.413)	0.061
Age	1.034	(1.009–1.060)	0.008
Mean LDL-c	2.658	(1.495–4.729)	0.001
Mean HDL-c	0.132	(0.016–1.073)	0.058
Model 2	** **	** **	** **
** **	OR	95% CI	*p*-Value
Sex	1.880	(1.000–3.532)	0.050
Age	1.040	(1.014–1.067)	0.003
Causative mutation	1.713	(0.902–3.256)	0.100
Mean LDL-c	2.336	(1.298–4.205)	0.005
Mean HDL-c	0.148	(0.018–1.223)	0.076
Model 3	** **	** **	** **
** **	OR	95% CI	*p*-Value
Sex	2.003	(1.076–3.728)	0.028
Age	1.044	(1.018–1.070)	0.001
Causative mutation	2.149	(1.170–3.948)	0.014
Mean HDL-c	0.236	(0.033–1.697)	0.151

OR, odds ratio; CI, confidence interval; LDL-c, low-density lipoprotein cholesterol; HDL-c, high-density lipoprotein cholesterol.

**Table 4:** Kruskal–Wallis test comparing the means of lipid panel components between study population sub-groups.

	No causative mutation (*N* = 167)	*LDLR* (*N* = 97)	Apo B (*N* = 22)	PCSK9/Apo E (*N* = 3)	*p*-Value
Total cholesterol (mg/dl)	287 ± 42	297 ± 68	309 ± 55	289 ± 77	0.551
LDL-c (mg/dl)	203 ± 37	225 ± 61	231 ± 52	218 ± 51	0.014
HDL-c (mg/dl)	63 ± 30	55 ± 14	63 ± 15	64 ± 14	0.017
Lpa (mg/dl)	46 ± 52	42 ± 46	37 ± 38	36 ± 34	0.986
Apo A1 (mg/dl)	159 ± 23	149 ± 26	159 ± 26	174 ± 41	0.033
Apo B (mg/dl)	146 ± 29	153 ± 42	162 ± 30	194 ± 81	0.150

LDL-R, low-density lipoprotein cholesterol receptor; PCSK9, proprotein convertase subtilisin/kexin type-9; Apo, apolipoprotein; LDL-c, low-density lipoprotein cholesterol; HDL-c, high-density lipoprotein cholesterol; Lpa, lipoprotein a.

**Table 5:** Bonferroni test comparing the mean difference of low-density lipoprotein cholesterol between study sub-groups.

		95% CI	*p*-Value
	*LDLR*	(−0.3733; −0.0506)	0.003
No mutation	PCSK9/Apo E	(−0.8827; 0.5895)	1
	Apo B	(−0.5645; 0.0086)	0.063
	No mutation	(0.0506; 0.3733)	0.003
*LDLR*	PCSK9/Apo E	(−0.6753; 0.8062)	1
	Apo B	(−0.3643; 0.2324)	1
	No mutation	(−0.5895; 0.8,827)	1
PCSK9/Apo E	*LDLR*	(−0.8062; 0.6753)	1
	Apo B	(−0.9091; 0.6463)	1
	No mutation	(−0.0086; 0.5645)	0.063
Apo B	*LDLR*	(−0.2324; 0.3643)	1
	PCSK9/Apo E	(−0.6463; 0.9091)	1

*LDLR*, low-density lipoprotein cholesterol receptor; PCSK9, proprotein convertase subtilisin/kexin type-9; Apo, apolipoprotein.

## Discussion

The present study is one of the few available studies to report on the prediction of HFH-causative variants including *APOE* variant type. It showed that HFH-causative variant carriers are more likely exposed to cardiovascular events and expressed a higher level of LDL-c, especially those with *LDLR* variant type. However, the detection of HFH-causative variant *per se* was not significantly associated with the occurrence of ASCVD in the course of time. Thus, the increased level of LDL-c remains the strongest independent predictor of ASCVD.

To date, available evidence on the effect of genetic variants on cardiovascular risk in HFH patients is controversial. For example, in Dutch HFH patients, the effect of *LDLR* variant type on survival analysis for freedom of cardiovascular event was only observed in the statistical models after excluding LDL-c level (11). Like us, authors conclude to a greater role of LDL-c level than causative variant *per se* on predicting cardiovascular risk. This shared conclusion ensues from the similarity between both study’s findings, in particular multivariable analysis results. Also, a recently published large French cohort has indirectly illustrated the same finding. This cohort has showed almost similar all-cause mortality rate in HFH patients with clinical versus genetic diagnosis (5.54 vs. 4.66 per 1,000 persons). Therefore, the rates of coronary events (24.66 vs. 15.89 per 1,000), cerebral events (3.44 vs. 2.47 per 1,000), and peripheral artery disease (3.63 vs. 2.66 per 1,000) were slightly higher in those with clinical diagnosis (12). In Copenhagen general population, no significant differences in coronary artery disease and myocardial infarction-free survival were observed between *APOB* variant carriers versus non-carriers (13). A significant difference in LDL-c level was mainly observed in patients expressing *LDLR* variant type (13,14). In opposition, some studies found that HFH patients with genetic variant are at elevated risk for adverse cardiovascular outcomes compared with no-variant group (15–18). Benn M. et al. reported a risk of coronary artery disease in LDL-c receptor gene mutation carriers 3.3 times higher than that in non-carriers (13). Khera and co-workers reported that being aware of FH mutation provides additional benefits on cardiovascular risk prediction than LDL-c level alone (17). Data from Japan suggest that genetic diagnosis may identify individuals at high risk by reflecting a lifetime exposure to the increased level of LDL-c (18). In France, we observed that one-third of patients carrying a severe mutation experienced a cardiovascular event with an average of 2.5 events per patient, while one-fourth of patients carrying a moderate mutation experienced a cardiovascular event with an average of two events per patient (19). A 2- to 3-fold increase in the risk of coronary artery disease has also been reported in HFH variant carriers (16). Indeed, the baseline LDL-c before initiating a medical treatment has been only used (16). In the present study, we were interested in LDL-c profile under optimal medical therapy as it may reflect more precisely the atherosclerotic impact of a causative variant in the real-world practice. Other studies have identified an association between *LDLR* variant type and ASCVD by revealing a link with carotid plaque formation (14) and obstructive coronary artery disease (13, 14, 17, 20). This augmentation in cardiovascular risk was not observed with the remaining genetic variants, *PCSK9* and *APOB*. In line with previously published studies, we do not reveal a significant difference in cardiovascular risk related to gender in HFH patients. However, it seems likely that males could be at higher risk and HDL-c level could be inversely associated with ASCVD in such HFH-treated population (21–23). Also, it is worth highlighting the potential role of non-LDL genetic factors that result in hypercoagulation and hypofibrinolysis as causal components of ASCVD in HFH patients, independent of elevated LDL-c (24, 25). For example, Kastelein’s group has shown an association between coagulation gene polymorphisms, e.g., G20210A, and ASCVD in FH patients (26). These investigators showed that FH individuals had increased factor VIII compared with non-FH (27). A literature review of these findings has been provided by Ravnskov et al. (28, 29). These findings may partially explain the beneficial effects of statins in FH due to their pleiotropic and anticoagulant effects (30–32). Thus, the discrepancy between the study’s results on atherosclerosis risk prediction of causative variant in HFH patients may be related to differences in non-LDL genetic factors’ expression among the study’s populations.

To summarize, it seems that the effect of a causative variant on atherosclerosis in HFH patients solely passes via the LDL-c level. Then, DNA analysis mainly plays a key role in the diagnosis and screening cascade. It provides an early diagnosis among family members and may reduce the lifelong exposure to high LDL-c level, whereas its usefulness for risk stratification remains uncertain (33, 34). A recently published paper highlights that the risk of incident cardiovascular disease event depends on a cumulative exposure to LDL-c (35). Otherwise, the cost–benefit analysis of genetic analysis tests is another concern even in the developed countries like Europe and Australia (36–38). Lastly, non-LDL genetic factors that result in hypercoagulation and hypofibrinolysis play a potential role as causal components of ASCVD in HFH patients, independent of elevated LDL-c.

## Limitations

The study design may predispose to selection bias. This study was carried out in a single large tertiary center, but this also promotes the homogeneity of the patient’s management and follow-up approach. The differences in lipid-lowering therapy and dose changes over time were not discussed. A large proportion of study participants were statin-intolerant patients. Statin intolerance is defined as the inability to tolerate at least two statins, one at the lowest starting dose. However, we assessed the last medical treatment of each of the study’s participants. We observed that 60.9% of study participants were treated with PCSK9i alone; 26.6% with PCSK9i and statins; 9.7% with PCSK9i, statins, and ezetimibe; and 2.8% with PCSK9i and ezetimibe. The number of collected lipid panel tests varies between study participants. In addition, we mention the small sample size and limited number of study participants in *PCSK9* and *APOE* sub-groups reducing the ability to make conclusion about differences among study sub-groups. The polygenic risk score in HFH-no causative variant carriers was not evaluated noticing that it is not yet widely performed due to a less robust evidence base for utility (39).

## Conclusion

The present study emphasizes the undisputable causal role of LDL-c for the occurrence of ischemic cardiovascular events in HFH patients with and without the causative genetic variant. While the incidence of ASCVD and level of LDL-c were higher in HFH pathogenic or likely pathogenic variant carriers, the detection of a causative variant did not represent *per se* an independent predictor of adverse cardiovascular outcomes. Thus, the usefulness of DNA analysis on top of LDL-c level for prognostic classification is uncertain. Additional larger prospective studies are warranted to examine this question.

## Data Availability

The raw data supporting the conclusions of this article will be made available by the authors, without undue reservation.
