# Supplementary material for: Effect of causative genetic variants on atherosclerotic cardiovascular disease in heterozygous familial hypercholesterolemia patients
Source: Front Cardiovasc Med. 2023 Jul 19;10:1182554. doi: 10.3389/fcvm.2023.1182554 (PMC10395089; doi:10.3389/fcvm.2023.1182554)
Supplement: Supplementary file 1 [file Table1.docx]

Supplementary materials:

Table1. Characteristics of study population without versus with moderate and severe causative-mutation.

|  | **Study population**  **(N=289)** | **Causative mutation-group (N=122)**  **Severe(N=36) Moderate(N=86)** | | **No causative mutation-group (N=167)** | **p-value** |
| --- | --- | --- | --- | --- | --- |
| **Age (Year)** | 49±13 | 43±13 | 45±15 | 52±11 | 0.001 |
| **Males (N,%)** | 107(37%) | 14(38.9%) | 32(37.2%) | 61(36.5%) | 0.961 |
| **BMI (kg/m²)** | 24.32±4.46 | 24.67±4.62 | 23.86±5.26 | 24.48±3.97 | 0.398 |
| **Smoker (N,%)** | 53(18.3%) | 6(16.7%) | 19(20.9%) | 29(17.4%) | 0.756 |
| **Systemic hypertension** | 44(15.2%) | 1(2.8%) | 13(15.1%) | 30(18%) | 0.071 |
| **Diabetes mellitus** | 8(2.8%) | 0(0%) | 4(4.7%) | 4(2.4%) | 0.442 |
| **Mean Total cholesterol (mg/dl)** | 292±54 | 284±45 | 306±72 | 288±42 | 0.022 |
| **Mean LDL-c (mg/dl)** | 213±49 | 217±44 | 229±64 | 203±37 | 0.001 |
| **Mean HDL-c (mg/dl)** | 60±25 | 53±11 | 58±16 | 63±30 | 0.026 |
| **Mean triglyceride (mg/dl)** | 125±55 | 102±38 | 113±42 | 137±61 | 0.001 |
| **Mean Lpa (mg/dl)**  **‹10mg/dl (%)**  **10-50mg/dl (%)**  **›50mg/dl (%)** | 44±49    33%  33%  34% | 52±57  29.6%  33.3%  37% | 36±36  33.8%  38.2%  27.9% | 46±52  33.3%  30.4%  36.3% | 0.256  0.532 |
| **Mean Apo A1 (mg/dl)** | 156±25 | 144±27 | 155±25 | 159±23 | 0.023 |
| **Mean Apo B (mg/dl)** | 150±35 | 147±38 | 160±42 | 146±29 | 0.017 |
| **ASCVD (N,%)** | 62(21.5%) | 10(27.8%) | 23(27.1%) | 29(17.4%) | 0.130 |
| **Follow-up (year)** | 5.97±5.97 | 8±5.6 | 6.96±6.89 | 4.98±5.28 | 0.003 |

Table 2. Statistical models of multivariable logistic regression investigating the association between the presence of causative mutation and development of atherosclerotic cardiovascular disease adjusted on confounders.

Model1

|  | OR | 95%CI | p-value |
| --- | --- | --- | --- |
| Age | 1.030 | [1.005-1.056] | 0.020 |
| Sex | 1.813 | [0.972-3.383] | 0.061 |
| Mean HDL | 0.167 | [0.021-1.346] | 0.093 |
| Mean LDL | 2.399 | [1.342-4.288] | 0.003 |
| Mutation  Moderate  Severe | 1.624  1.698 | [0.806-3.273]  [0.674-4.276] | 0.314  0.175  0.261 |

Model 2

|  | OR | 95%CI | p-value |
| --- | --- | --- | --- |
| Age | 1.033 | [1.008-1.058] | 0.009 |
| Sex | 1.924 | [1.041-3.555] | 0.037 |
| Mean HDL | 0.256 | [0.036-1.825] | 0.174 |
| Mutation  Moderate  Severe | 2.134  1.975 | [1.105-4.121]  [0.798-4.890] | 0.024  0.141 |
